# Comparing the feasibility, acceptability, clinical-, and cost-effectiveness of mental health e-screening to paper-based screening on the detection of depression, anxiety, and psychosocial risk in pregnant women: a study protocol of a randomized, parallel-group, superiority trial

**DOI:** 10.1186/1745-6215-15-3

**Published:** 2014-01-02

**Authors:** Dawn Kingston, Sheila McDonald, Anne Biringer, Marie-Paule Austin, Kathy Hegadoren, Sarah McDonald, Rebecca Giallo, Arto Ohinmaa, Gerri Lasiuk, Glenda MacQueen, Wendy Sword, Marie Lane-Smith, Sander Veldhuyzen van Zanten

**Affiliations:** 1 University of Alberta, 11405-87th Avenue, Edmonton, T6G 1C9, Canada; 2 University of Calgary, Calgary, AB T2N 1 N4, Canada; 3 University of Toronto, Toronto, ON, Canada; 4 University of New South Wales (AU), Kensington NSW 2052, Australia; 5 McMaster University, Hamilton, ON L8S 4 L8, Canada; 6 Parenting Research Centre, East Melbourne, VIC 3002, Australia

**Keywords:** Psychosocial assessment, Online, Screening, Pregnancy, Depression, Anxiety, Stress, Randomized controlled trial

## Abstract

**Background:**

Stress, depression, and anxiety affect 15% to 25% of pregnant women. However, substantial barriers to psychosocial assessment exist, resulting in less than 20% of prenatal care providers assessing and treating mental health problems. Moreover, pregnant women are often reluctant to disclose their mental health concerns to a healthcare provider. Identifying screening and assessment tools and procedures that are acceptable to both women and service providers, cost-effective, and clinically useful is needed.

**Methods/Design:**

The primary objective of this randomized, parallel-group, superiority trial is to evaluate the feasibility and acceptability of a computer tablet-based prenatal psychosocial assessment (e-screening) compared to paper-based screening. Secondary objectives are to compare the two modes of screening on: (1) the level of detection of prenatal depression and anxiety symptoms and psychosocial risk; (2) the level of disclosure of symptoms; (3) the factors associated with feasibility, acceptability, and disclosure; (4) the psychometric properties of the e-version of the assessment tools; and (5) cost-effectiveness. A sample of 542 women will be recruited from large, primary care maternity clinics and a high-risk antenatal unit in an urban Canadian city. Pregnant women are eligible to participate if they: (1) receive care at one of the recruitment sites; (2) are able to speak/read English; (3) are willing to be randomized to e-screening; and (4) are willing to participate in a follow-up diagnostic interview within 1 week of recruitment. Allocation is by computer-generated randomization. Women in the intervention group will complete an online psychosocial assessment on a computer tablet, while those in the control group will complete the same assessment in paper-based form. All women will complete baseline questionnaires at the time of recruitment and will participate in a diagnostic interview within 1 week of recruitment. Research assistants conducting diagnostic interviews and physicians will be blinded. A qualitative descriptive study involving healthcare providers from the recruitment sites and women will provide data on feasibility and acceptability of the intervention. We hypothesize that mental health e-screening in primary care maternity settings and high-risk antenatal units will be as or more feasible, acceptable, and capable of detecting depression, anxiety, and psychosocial risk compared to paper-based screening.

**Trial registration:**

ClinicalTrials.gov Identifier: NCT01899534.

## Background

### Introduction

Depression and anxiety are among the most common morbidities in pregnancy and postpartum (up to 1 year post-delivery), with prevalence rates of 13% to 29% [1-3]. Without early screening and treatment, 50% to 70% of women with prenatal anxiety or depression symptoms [4] will experience persistent symptoms through their child’s early years [5,6] with enduring effects on their children’s development and mental health [7-9]. Psychiatric illness is also the leading cause of maternal mortality in Western countries [10]. However, perinatal mental health problems are severely underdetected and undertreated [11,12]. Without standardized screening, 80% of cases remain undetected [13,14].

While screening is important, there are significant barriers that prevent the majority of pregnant and postpartum women from seeking mental healthcare and disclosing concerns. These include the stigma of mental health, lack of understanding of whether symptoms are abnormal or a typical pregnancy experience, having providers or support persons underestimate their symptoms and concerns, and fear that reporting symptoms will lead others to think that they will be an incompetent mother, prevent the majority of pregnant and postpartum women from seeking mental healthcare and disclosing concerns [15-19]. Although such barriers may prevent women from accessing support on their own, only 4% of women refuse mental health screening when offered by healthcare providers [20,21].

Despite recommendations [22,23] and high acceptance by women [24-26] and providers [27-30], only 20% of perinatal providers conduct proactive screening as part of prenatal care [31] and less than 15% of pregnant/postpartum women receive the help they need [32]. A systematic review conducted by our team (manuscript in preparation) found that substantial personal and system barriers to routine screening exist for healthcare providers, including fear of women’s responses to screening, lack of time, lack of accurate assessment tools and knowledge of their interpretation, and lack of referral processes and options [20,33,34].

Taken together, this body of research underscores the need to identify screening and assessment tools and procedures that are acceptable to both women and service providers, overcome barriers to implementation, and are cost-effective and clinically useful. The most effective perinatal mental health screening and management programs are those characterized by screening incorporated into routine care with designated systems of referral and treatment that are initiated immediately after screening [35].

#### *The potential impact of e-screening*

Routine, standardized screening significantly improves detection of mental health problems [13,14]. However, the scarcity of human health resources poses a major deterrent to routine screening. E-screening has the potential to increase efficiency of mental healthcare by reallocating limited human resources where they are most needed - in-depth follow-up assessment, referral, and treatment. It is a low-resource option [36,37] that can be embedded in current prenatal and postpartum care across various settings and providers (for example, midwives, nurses, obstetricians, family physicians) and thus will increase access to routine screening.

Importantly, e-screening can address the most prominent barriers to screening identified by pregnant women and healthcare providers. Several studies report that e-screening for sensitive issues such as prenatal/postnatal intimate partner violence [38-40] and postpartum depression [41] is acceptable and feasible. It is well-suited for busy clinical settings in that it offers consistency, can be tailored to patient needs, can be used with audio/video for low literacy, provides real-time data [36,37], achieves similar or greater rates of disclosure compared to interviews, and is preferred by patients due to its anonymity [37,38,42,43]. To date, no studies have evaluated e-screening in pregnant women, yet screening that increases access to early prenatal intervention may reduce the risk of prenatal and postpartum depression/anxiety, as well as adverse child outcomes. One small study that examined the psychometric properties and rates of detection of postpartum depression of two Internet-based screening tools found that both tools (Edinburgh Postnatal Depression Scale; The Postpartum Depression Screening Scale) had excellent validity and reliability with prevalence rates within range of other self-report and interview-based assessment approaches [41].

There is a clear need for a rigorous evaluation of the feasibility, acceptability, and psychometric performance of e-screening for prenatal mental health difficulties. A key consideration for evaluation is to determine whether established assessment tools are valid and reliable for use when delivered online. For example, it has been shown that some tools have different psychometric properties when delivered online, suggesting a need for different cutoff points [44]. Another key issue is the inclusion of women experiencing a broad range of degrees of psychosocial risk. Pregnant women with high-risk pregnancies represent a vulnerable group in that the prevalence of mental health problems in this group is more than three times greater than medically low-risk women [45,46]; however, medically high-risk women are rarely included in intervention studies of perinatal mental health.

#### *Screening versus psychosocial assessment*

Screening is defined as the use of a symptom-based tool, and psychosocial assessment is the combined use of a screening tool plus an assessment of psychosocial risk factors [3]. In this protocol, e-screening refers to use of the Internet to collect and transfer data from a psychosocial assessment.

## Purpose

### Objectives, research questions, and hypotheses

#### *Primary objective*

The primary objective of the study is to determine pregnant women’s and healthcare providers’ views of the feasibility and acceptability of mental health e-screening compared to paper-based screening.

#### *Secondary objectives*

The five secondary objectives are to compare the two modes of screening on: (1) the level of detection of prenatal depression and anxiety symptoms and psychosocial risk; (2) the level of disclosure of symptoms; (3) the factors associated with the acceptability, feasibility, and disclosure; (4) psychometric properties (sensitivity, specificity, positive and negative predictive values) of the e-version of the Antenatal Psychosocial Health Assessment (ALPHA) and Edinburgh Postnatal Depression Scale (EPDS) when administered to medically low- and high-risk pregnant women; and (5) cost-effectiveness of screening.

### Research questions and hypotheses

The specific research questions and hypotheses corresponding to the primary and secondary objectives are described in Table 1. Overall, we hypothesize that mental health e-screening in primary care maternity settings and high-risk antenatal units will be as or more feasible, acceptable, and capable of detecting depression, anxiety, and psychosocial risk compared to paper-based screening.

**Table 1:** Primary and secondary objectives, research questions, and hypotheses

**a**				
**Primary objective**	**Research question**	**Outcome**	**Measures**	**Testable hypothesis**
To compare the feasibility and acceptability of mental health e-screening *versus* paper-based screening.	Is mental health e-screening as or more feasible and acceptable to pregnant women and their healthcare providers than paper-based screening?	Feasibility: % women in intervention and control groups reporting that screening is easily done as a component of routine prenatal care; mean CAE score	Quantitative:	Mean CAE scores and % of women responding affirmatively to questions of feasibility and acceptability will be similar or significantly higher in the intervention group (indicating greater feasibility/acceptability) compared to the control group
			Feasibility	
			Example (CAE): I liked/would like using the tablet to answer these questions	
		Acceptability: % of participants in intervention and control groups reporting that screening is acceptable; % of participants reporting that questions about emotional health were easy to understand and easy to navigate around on the tablet	Acceptability	
			Example: (1) I did not/would not like answering questions on a tablet because it felt/would feel impersonal	
			Qualitative:	
			Semi-structured interviews	
**b**				
**Secondary objective**	**Research question**	**Outcome**	**Measure**	**Testable hypotheses**
1. To compare the level of detection of symptoms of prenatal depression, anxiety, and psychosocial risk in e-screening *versus* paper-based screening	Compared to paper-based screening, what is the effect of mental health e-screening in pregnant women on the detection of prenatal depression, anxiety, and psychosocial risk?	Proportion of women scoring above cutoff point of EPDS for depression and anxiety; proportion of women identified as some or high psychosocial risk on ALPHA	EPDS	Compared to the control group, a higher proportion of women in the intervention group will: (1) score 13 or more on the total EPDS (corresponding to probable prenatal depression); (2) score 4 or more on the anxiety subscale of the EPDS (Q3, 4, 5) (corresponding to probable prenatal anxiety); and (3) be identified as having some/moderate or high psychosocial risk on the ALPHA
			ALPHA	
2. To compare the level of disclosure of symptoms of prenatal depression and anxiety, and psychosocial risk in e-screening *versus* paper-based screening	Compared to paper-based screening, what is the effect of mental health e-screening in pregnant women on the disclosure of prenatal depression, anxiety, and psychosocial risk?	Level of disclosure: Mean subscale scores: (1) risk of disclosure; (2) benefits of disclosure	DES	Compared to paper-based screening, e-screening promotes greater disclosure (for example, the mean score risk of disclosure is significantly lower and benefit of disclosure is significantly higher in the e-screening group)
3. To determine factors associated with the acceptability and feasibility of mental health e-screening as well as disclosure	What factors are associated with acceptability and feasibility of mental health e-screening in pregnant women?	Identification of factors that significantly increase odds of acceptability and feasibility of e-screening	Quantitative: (1) Demographic variables (age, gestation, marital status; ethnicity); (2) mental health history; current mental health status; EPDS scores; ALPHA category; (3) DES scores; (4) medical risk; (5) features of the screening instrument/process	Factors that are significantly associated with acceptability and feasibility: mental health history, current mental health status (EPDS, ALPHA), disclosure (DES), medical risk; features of screening instrument
			Qualitative:	
			Semi-structured interviews	
				Factors not associated: demographics
4. To compare the psychometric properties (sensitivity, specificity, cutoff points) of paper-based ALPHA and EPDS *versus* the e-version administered to medically low- and high-risk pregnant women	Are the psychometric properties (for example, sensitivity, specificity, cutoff points) similar or better in the ALPHA and EPDS e-version compared to the paper-version when administered to pregnant women?	Psychometric properties: sensitivity, specificity of ALPHA and EPDS in paper-based and e-versions	ALPHA, EPDS, MINI	The psychometric properties of e-version of ALPHA and EPDS are similar or better compared to paper-based version
5. To compare the cost-effectiveness e-screening compared to paper-based screening	Is e-screening as a component of routine prenatal care cost effective when compared to paper-based screening?	Cost-effectiveness: actual costs	The expected incremental cost effectiveness of e-screening is cost effective at values of health considered acceptable in the Canadian healthcare system	E-screening will be cost effective

ALPHA: Antenatal Psychosocial Health Assessment; CAE: CASI Assessment Evaluation; DES: Disclosure Expectations Scale; EPDS: Edinburgh Postnatal Depression Scale; MINI: Mini International Neuropsychiatric Interview.

## Methods/Design

### Study design

**Figure 1:**
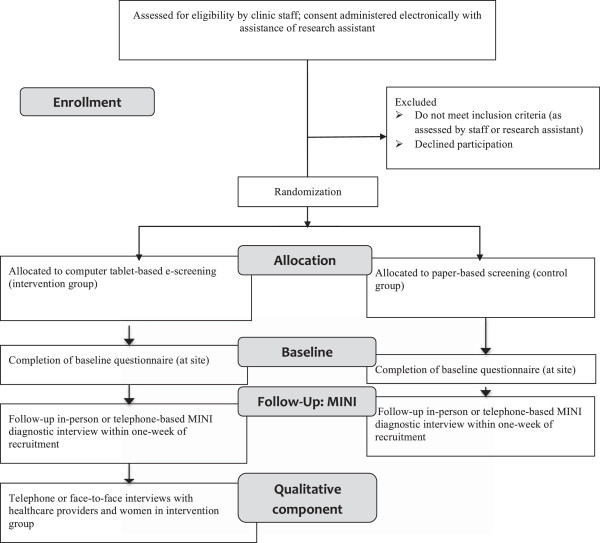
**CONSORT trial flow diagram.** *ALPHA: Antenatal Psychosocial Health Assessment; EPDS: Edinburgh Postnatal Depression Scale; MINI: Mini International Neuropsychiatric Interview.

The study is a parallel-group, randomized, controlled superiority trial (RCT) with a qualitative descriptive component (Figure 1). This design is well-suited for answering questions of effectiveness, acceptability, and feasibility [47]. In this trial, physicians and all research assistants conducting diagnostic interviews are blinded. We adhered to CONSORT guidelines in the design of the trial [45,46] and the SPIRIT guidelines [48,49] in reporting its details in this protocol. Approval for this study was granted by the Human Research Ethics Board at the University of Alberta.

### Randomized controlled trial

#### *Eligibility criteria*

Pregnant women are eligible for this study if they: (1) receive care at one of the recruitment sites; (2) are able to speak/read English; (3) are willing to be randomized to e-screening; and (4) are willing to participate in a follow-up diagnostic interview within 1 week of recruitment.

#### *Setting*

Recruitment of pregnant women will take place at maternity clinics in a large, urban Canadian city and an inpatient, high-risk antenatal unit in a tertiary care hospital. Medically high-risk pregnant women have significantly higher rates of depression and anxiety than low risk women [45,46]. Thus, recruiting women with low and high medical risk will enable us to evaluate the effectiveness of e-screening to accurately detect symptoms of depression, anxiety, and psychosocial risk across the spectrum of low to high symptom prevalence and severity. All sites serve a sociodemographically diverse population. At the maternity clinics, family physicians specializing in obstetrics provide full obstetrical care (for example, all prenatal, delivery, and postpartum care) and shared care (for example, they provide prenatal care up to 28 weeks and then care is transferred to an obstetrician). The high-risk antenatal unit is a 24-bed inpatient unit in a large, tertiary care hospital with over 5,900 births per year. It is the only inpatient acute hospital in the community and surrounding region for women with high-risk pregnancies, and women receive care primarily from obstetricians. The majority of family physicians, obstetricians, and nurses at the recruitment sites do not have specialized training in mental healthcare.

#### *Recruitment procedures, consent, randomization, and allocation procedures*

Recruitment procedures are similar across sites. Trained staff at the recruitment sites will use a standardized script to determine each eligible (see Eligibility criteria) woman’s interest in study participation. Women will be referred to an onsite research assistant who will provide the tablet. Women will complete the consent electronically on the tablet and receive the automatically generated copy via email. The research assistant will be available to answer questions about study participation and tablet logistics. A simple, computer-generated randomization process designed by the Women’s and Children’s Health Research Institute (WCHRI) at the University of Alberta allocates women (1:1 ratio) automatically to the control or intervention group, and they will receive a ‘pop-up’ message informing them that they will complete questions about emotional health on paper (control group) or tablet (intervention group). Computerized randomization preserves allocation concealment and reduces the possibility of selection bias since the research assistant is kept unaware of the group assignments until after the participants are allocated to groups.

#### *The intervention*

The intervention is psychosocial assessment via electronic administration. Women assigned to the intervention group will complete the psychosocial assessment comprising the ALPHA and the EPDS electronically using a computer tablet. Rather than the traditional, sole use of symptom-based mental health screening tools, the combined use of a psychosocial risk assessment instrument (for example, ALPHA) that detects current and future risk for mental health problems and a symptom screening tool (for example, EPDS) that detects current symptomatology has been recommended as the most effective, comprehensive approach to psychosocial assessment [3].

The ALPHA was selected for use as a psychosocial risk assessment tool because its paper-based version is currently used in our clinic recruitment sites. In addition, the ALPHA is a Canadian-developed tool that has been tested in the Canadian context in primary care [13,30] and has been gaining popularity for antenatal psychosocial assessment in these settings. It was designed to identify psychosocial risk factors in pregnant women that may increase their risk for adverse psychosocial outcomes (for example, stressful life events, lack of support), including postpartum depression [50,51]. It has undergone extensive face and content validity by experts including obstetricians, family physicians, and midwives [30], and identifies significantly more cases of psychosocial risk than non-standardized assessment [51]. The majority of providers indicated that the tool could readily be implemented in routine prenatal care and pregnant women found it acceptable [33]. The self-report version used in this study is easily completed in 5 to 10 minutes. It asks women questions on the topics of family life, stressors, feelings about the pregnancy, substance use, abuse, and family of origin. Women respond with yes/no or 6-point Likert-scale options. Based on a review of responses in each category, trained providers subjectively judge whether women are at low, some, or high psychosocial risk. No cutoff points or scoring algorithm have been developed and thus sensitivity and specificity data are not available. Risk categories are then used to create a care plan.

The 10-item EPDS was selected for evaluation because it is one of the most widely used screening instruments for postpartum, and less commonly, prenatal depression. The EPDS was designed as a brief tool to screen for postpartum depression symptoms within the previous 7 days [52]. A score of 13 or greater (range, 0 to 30) is a well-established cutoff for clinically significant depression symptoms consistent with meeting criteria for a major depressive episode [52]. Original psychometric testing resulted in sensitivity of 85%, specificity of 77%, the positive predictive value of 83%, split-half reliability of 0.8, and a Cronbach’s alpha of 0.87 [52]. The 3-item anxiety subscale of the EPDS is a reliable and valid tool for probable anxiety using a cutoff of 4 or more with a Cronbach’s alpha of 0.74, sensitivity of 66.7%, and specificity of 73.2% [35,53].

#### *The control group*

Women in the control group will complete paper-based versions of the ALPHA and EPDS. Usual prenatal care at our clinic recruitment sites involves women self-completing the ALPHA on a single occasion during pregnancy, typically while they wait for their first or second prenatal visit. At the hospital recruitment site, usual care does not include formal screening, although women’s mental health status is monitored due to the high medical risk. Paper-based screening was chosen as the comparator because the primary objective of the study is to assess feasibility and acceptability of the electronic administration of a psychosocial assessment.

#### *Procedures*

##### 

**Maternity clinics** At the maternity clinic recruitment sites, each eligible woman will complete the ALPHA and EPDS while they wait for their prenatal appointment - either via computer tablet or paper. After randomization to the control group, a message appears on the tablet, directing women to complete the ALPHA and EPDS on paper. An automatic ‘skip’ procedure ensures that the electronic versions of the ALPHA and EPDS are bypassed for women in the control group. Following completion of the paper-based psychosocial assessment, women will proceed to complete the baseline questionnaire on the tablet. Women in the intervention group will be directed to answer questions about emotional health (ALPHA and EPDS) and the baseline questionnaire on the tablet. All questions require responses, and thus women cannot proceed past questions with missing answers until completed. In keeping with the current clinic processes of including the assessment in the medical record, the electronic copy will be printed following completion. As is usual care in the maternity clinics, a Registered Nurse will review the assessment, judge whether women are at low, some, or high risk, discuss women’s psychosocial assessment responses with them, and provide follow-up referrals for those with some or high psychosocial risk with a mental health nurse affiliated with the clinic.

##### 

**Hospital-based antenatal unit** In the antenatal unit, the research assistant will meet with women who have agreed to a follow-up contact by the research team. The research assistant will provide the tablet, a copy of the paper-based questionnaire, and an opaque envelope. The research assistant will be available to answer questions related to study participation, but then will leave the room to allow the woman to proceed to the computer randomization, and complete the assessment and baseline questionnaire independently. Thus, the research assistant will have no knowledge of the woman’s group assignment. Following randomization to the intervention group, women will be directed by a computer message to complete the ALPHA and EPDS on the tablet as well as the baseline questionnaire. Women assigned to the control group will be directed by a computer message to complete the ALPHA and EPDS on paper and then return to the tablet for completion of the baseline questionnaire. An automatic ‘skip’ procedure ensures that the electronic versions of the ALPHA and EPDS are bypassed for women in the control group.

In both cases, prior to the final submission of the baseline questionnaire, intervention and control group women will be prompted to place the paper-version questionnaire in the opaque envelope and seal it.

In the antenatal unit setting, procedures to maintain blinding of the research assistant are required because he/she will be both recruiting women and conducting the follow-up diagnostic interview. Blinding of the research assistant is maintained by: (1) distributing an opaque envelope and a paper-based version of the ALPHA and EPDS to all participants prior to randomization; (2) using a computer-based randomization process that reveals the assignment to the woman, but not the research assistant; (3) instructing all women to return the paper-based assessments to the envelope prior to giving the tablet and envelope back to the research assistant (an electronic message on the tablet prompts women to return the paper-based assessment to the envelope); and (4) training the research assistant to return all envelopes (unopened) to the research coordinator.

Two processes have been designed to ensure that the correct versions of the ALPHA and EPDS are completed (for example, paper *versus* electronic) and to avert the possibility that participants might self-select a preferred version: (1) the electronic versions of the ALPHA and EPDS are not available to women in the control group through a programmed ‘skip’ procedure; and (2) the research coordinator will conduct a manual check on a subset of 30 control group participants at three different times in the course of the study to ensure that paper-based versions of the ALPHA and EPDS are completed.

If women are identified as meeting criteria for a mood or anxiety disorder on the MINI International Neuropsychiatric Interview (see Confirmation of diagnosis) or if they score 13 or more on the EPDS the research assistant will create a referral for the woman (with her permission) to the hospital-based reproductive mental health support program and she will be followed up by a mental health therapist.

#### *Confirmation of diagnosis*

All women will have a MINI International Neuropsychiatric Interview (MINI, Version 6.0.0) by a trained, blinded research team member skilled in diagnostic interviews within 1 week of completing their psychosocial assessment. The MINI is a brief, ‘gold standard’ diagnostic interview designed to assess the presence of mental disorders according to criteria defined in the Diagnostic and Statistical Manual of Mental Disorders IV (DSM-IV) [49]. It can be administered in approximately 15 minutes with interviewees providing yes/no responses. All research staff conducting diagnostic interviews will participate in four 3-hour training sessions on the structure and completion of the MINI, interviewing skills, approaches to asking sensitive questions, and role-playing. Additionally, the first two to three interviews will be supervised by one of the mental health clinicians on our team (KMH).

For women recruited through the maternity clinics, a research team member not involved in recruitment will conduct a telephone-based MINI within 1 week of the woman completing the psychosocial assessment (at the clinic). Because of space and time constraints in the maternity clinics, it is necessary to conduct the MINI via telephone, and follow-up by a different research assistant maintains blinding. For women recruited through the inpatient antenatal unit, the MINI will be conducted at the time of recruitment, after completion of the psychosocial assessment and baseline questionnaire. The research assistant involved in recruitment will conduct the MINI, with specific measures employed to maintain blinding (see Procedures).

#### *Outcomes and measurement*

##### 

**Primary outcome** The primary outcome is feasibility and acceptability of screening (for example, e-screening *versus* paper-based screening) (Table 1a).

##### 

**Feasibility** All women will complete the 10-item CASI Assessment Evaluation (CAE), an established instrument designed to evaluate the feasibility of computer-based screening [40]. Originally designed to assess computer-based screening of intimate-partner violence, the CAE’s questions are relevant to assess feasibility of e-screening for prenatal mental health, and its content is more extensive than measures used in other studies evaluating different screening modalities in pregnant/postpartum women [38]. The CAE has demonstrated face, construct, and discriminant validity, and provides a standardized assessment of feasibility of e-technology [40]. Women in the intervention group will answer questions rating their experience of e-screening. Women in the control group will answer questions adapted from the CAE to assess their views of e-screening (for example, ‘Would you have been comfortable using a computer tablet to answer these questions?’).

##### 

**Acceptability** Questions adapted from other surveys of mental health screening acceptability [24,25,54] will be administered to women in both control and intervention groups to evaluate acceptability of the form of screening they completed. In addition, we will use semi-structured face-to-face or telephone-based interviews to determine views of feasibility and acceptability among a subset of 20 participants and 8 healthcare providers.

### Secondary outcomes

The five secondary outcomes are (see measures in Table 1b):

(1) the proportion of pregnant women scoring 13 or more on the total EPDS, 4 or more on the anxiety subscale, and identified as some or high psychosocial risk on the ALPHA;

(2) the level of disclosure as assessed by mean scores on the Perceived Risk and Perceived Utility subscales of the Disclosure Expectations Scale (DES) [55]. The DES is an 8-item scale designed for evaluation of the perceived risk (items 1, 2, 4, and 5) and benefit (items 3, 6, 7, and 8) of disclosure. Convergent validity of the subscales has been demonstrated with other measures of self-disclosure, as well as psychological distress, and intention to seek mental healthcare [55];

(3) description of factors that significantly increase the odds of acceptability, feasibility, and disclosure related to each form of screening;

(4) psychometric properties (for example, Cronbach’s alpha, sensitivity, specificity, positive and negative predictive values) of the e-version of the EPDS and ALPHA. A scoring algorithm does not currently exist for the ALPHA (for example, the trained provider subjectively judges risk based on responses); however, an algorithm would promote standardization in clinical decision-making and efficiency since a score could be calculated as part of e-screening. We plan further tool development comprising derivation of a quantitative scoring method for classifying women into psychosocial risk categories; and

(5) cost-effectiveness of detecting clinical depression and anxiety in e-screening *versus* usual screening (that is, equipment and human resource costs of e-screening *versus* paper-based - see Secondary outcomes).

#### Sample size

##### 

**Determination of sample size** Because no data are available to guide estimation of a minimal clinically important difference in ‘true’ cases detected through e-screening a ‘confidence interval (CI) approach’ [56] was used. Based on high levels of acceptability and disclosure reported using computer screening [39,40], e-screening would be feasible if, in the intervention group: (1) 85% of women indicated that they were able to tell the truth on all items (Q7 CAE); (2) 85% scored 4 to 8 on the Risk subscale of the DES (no/slight perceived risk to disclosure); and (3) 85% scored 16 to 20 on the DES Utility subscale (for example, disclosure moderately or very beneficial). The sample size calculation (Table 2) indicates that 261 women per group (*n* = 522) is required.

**Table 2:** Sample size determination

**Using the calculation of a confidence interval for a proportion, a 95% CI and a margin of error of 0.05:**	**Accounting for an estimated attrition/loss to follow-up of 25% based on other reported rates in pregnant and postpartum women (55,66):**
p = a priori estimate of % of interest; *n* = sample size	*n* = total sample size; L = attrition rate + loss-to-follow up
*n* = p(1-p) [1.96/.05]^2^	N_new_ = n/(1-L)
*n* = 0.85(1–0.85) [39.2]^2^	N_new_ = 196/(1–0.25)
*n* = 196	N_new_ =261

Therefore a minimum of 261 women per group would be required.

##### 

**Feasibility of achieving sample size** The maternity clinics conduct initial prenatal visits for a total of 50 women per month across all clinics. The number of new patients on the high-risk antenatal unit is similar at 45 women per month. Clinic staff estimates that roughly 4% of women would be ineligible due to language (96% eligible = 91 women per month). A conservative estimation of 50% participation rate [57,58] would yield 46 women per month. Thus, recruitment of women is expected to take 12 months.

#### *Data collection procedures*

Procedures related to completion of the psychosocial assessment and baseline questionnaire are described in Procedures. Women will complete the psychosocial assessment on a single occasion. The content and order of individual questions in the e-version of the instruments will be identical to the paper-based version. Women select responses by touching the screen. If a woman does not answer a question, she will be prompted to respond before she can proceed. This is to mimic the usual screening process where the nurse would ask the woman to respond to a missing answer. A ‘SUBMIT’ button at the bottom of the baseline questionnaire will allow women to submit their responses.

The order of material on the tablet will be: (1) Participant Information Letter and Consent; (2) preamble describing the importance of screening and the follow-up with the healthcare provider; (3) ALPHA and EPDS; (4) the CAE, DES, and questions related to acceptability, feasibility, and disclosure; and (5) baseline questionnaire. The content of the baseline questionnaire includes questions that represent factors associated with prenatal mental health or disclosure of mental health concerns, and includes: (1) demographics (age, parity, marital status, education, income, ethnicity, country of birth, and length of time in Canada); (2) obstetrical history (current and past, including use of fertility treatments); (3) mental health history (diagnoses, treatment); (4) level of comfort with technology (for example, laptop, tablet); (5) quality of relationship with perinatal care provider; (6) level of social support, experience of talking with doctor/nurse/midwife about emotional health; and (7) adverse childhood experiences (using ACES questionnaire [48]). With the exception of the ACES (http://acestudy.org/ace_score), questions were derived from the All Our Babies longitudinal birth cohort study (http://www.prehot.org/The%20All%20Our%20Babies%20Study/) and the Maternity Experiences Survey (http://www.phac-aspc.gc.ca/rhs-ssg/survey-eng.php). Participants are required to answer all fields except for income, thus limiting the issue of missing data. Data for the economic evaluation will be collected from program accounts for technology and software costs, and by interviewing clinic staff to estimate clinic operation costs for the two alternatives. We will use a healthcare perspective and all costs will be shown in 2014 values.

No data will be stored on the tablets. When women ‘submit’ their information it will be sent to a secure server housed in the Faculty of Medicine & Dentistry’s Data Centre at the University of Alberta. The psychosocial assessment and questionnaire were built using an existing infrastructure offered by Checkbox Survey software provided by Women’s and Children’s Health Research Institute’s (WCHRI) Clinical Research Informatics. Data transfer between the tablet and server will be encrypted. Data imported to statistical databases for analysis will not be identifiable.

### Analysis

#### *Primary outcome and description of sample*

Intention-to-treat analysis will be conducted for all analyses. We will use descriptive data (frequencies/95% CI; means/standard deviation) to describe the sample. Baseline differences in groups will be compared using independent t-tests (means) and chi-square tests (%) to determine the extent to which randomization was successful. Statistical significance for all analyses is set at *P* <0.05. We will use two-tailed independent t-tests to compare intervention and control group differences in mean scores of the CAE and chi-square tests to compare proportions of women in each group responding affirmatively to questions on feasibility and acceptability.

#### *Secondary outcomes*

##### 

**Detection** Differences in proportions of women in the control and intervention group scoring 13 or more on the EPDS, and categorized as some or high-risk on the ALPHA will be estimated using chi-square tests.

##### 

**Disclosure** Comparison of mean scores of the subscales of the DES (risk, benefit of disclosure) will be done using two-tailed independent sample t-tests.

##### 

**Factors associated with acceptability, feasibility, and disclosure** We will use multivariable logistic regression to determine predictors of acceptability, feasibility, and disclosure and report relative risks and 95% CIs. Outcomes will be dichomotized measures of acceptability, feasibility (based on CAE), and disclosure (based on DES) where ‘high’ acceptability, feasibility, and disclosure will include participants whose responses place them above the 75th percentile for the measure. Models will be built using variables that are associated with outcomes at *P* <0.10 on crude analyses, entering demographic variables in a first block, followed by obstetrical factors, mental health history, and the ACES score. Results will be reported as odds ratios and 95% CIs.

##### 

**Psychometric properties** Psychometric properties of internal consistency (Cronbach’s alpha; item-total correlation) will be calculated for the EPDS by modality. Predictive validity measures of sensitivity, specificity, positive predictive value, and negative predictive value will be calculated using clinical interview diagnosis as the reference. Comparisons of the psychometrics between the two modalities will be made by correlational analysis using Cohen’s guidelines [59]. In terms of further tool development of the ALPHA, we will develop an integer score-based prediction rule for the prevalence of anxiety and depression, according to MINI diagnosis. This method was previously used to develop a prenatal screening tool for postpartum distress by two members of the research team (SM, DK) [60]. We will develop a best fit multiple regression model predicting depression and/or anxiety as per clinical interview diagnosis, candidate variables for which will be drawn from the ALPHA. A regression coefficient-based scoring algorithm [60] will be applied to the final regression model in order to develop a single screening score. Receiver operating characteristic (ROC) analysis will then be used to determine an optimal cutoff for the screening score. Finally, we will perform a ROC analysis for each modality (e-version *versus* paper-based) and compare indices of sensitivity, specificity, positive and negative predictive values using Cohen’s guidelines [60]. All analyses will be conducted in the total sample, with subgroup analyses conducted by medical risk to determine whether the scoring is robust across risk subgroups.

##### 

**Cost-effectiveness** For the economic analysis, we will estimate the incremental costs-effectiveness (ICER) for the e-screening alternative compared to the paper-based alternative. The e-screening costs include investment costs to technology (programs, tablets, data storage) and the operating costs at the clinic that may differ somewhat from the conventional group administrative and analytical cost. Cost-effectiveness is determined by incremental cost to identify a woman with increased risk of prenatal and postpartum depression and anxiety. Because we are not going to follow up the screened women, the costs and outcomes after the screening are not included in this initial economic analysis.

## Qualitative descriptive study

### Methods

#### *Participant eligibility and recruitment*

All women and healthcare providers working at the study sites are eligible for participation in the qualitative component. Purposeful sampling will be used to maximize variability in the sample, ensuring that a broad range of views and demographic (for example, income age, marital status, ethnicity), medical (for example, mental health history; current mental health status), and social (ACES) factors are represented [61]. We plan to interview approximately 20 women (10 intervention group, 10 control group) and eight to 10 providers (for example, nurses, family physicians) with the final sample size determined by data saturation. Women will be invited by the research assistant to participate in a follow-up qualitative interview during the diagnostic interview. Healthcare providers will be invited through emails distributed by the managers.

#### *Data collection and management*

We will conduct individual face-to-face and telephone-based interviews. Semi-structured interview guides will be used [61] to ask participants their views on the feasibility and acceptability of screening by e-screening or paper-based version (depending on group membership), as well as its strengths, suggestions for improvement, and the challenges and benefits they experienced. Interviews are expected to take 30 to 60 minutes and will be digitally recorded and transcribed verbatim. Transcribed interviews and digital files will be stored in the secure environment of the Health Research Data Repository at the University of Alberta. All data will be anonymized for publication. Only researchers affiliated with the study will have access to participant data.

#### *Analysis*

As recommended for qualitative descriptive studies, we will use standard qualitative content analysis approaches for thematic analysis of the transcripts [61]. Two members of the team experienced in qualitative data analysis will independently code the first two or three transcripts and attain consensus on a coding scheme. These two team members will use this coding scheme to analyze two to three more transcripts, and a revised coding scheme will be established as necessary. The well-developed coding scheme will then be refined to the point where one research team member can independently code the remaining transcripts, with revisions made as necessary to reflect new and evolving themes as data analysis progresses [61]. Thematic analysis will occur concurrently with data collection to allow further exploration and clarification of emergent ideas, and data collection will continue until data saturation is reached [62].

## Conclusion

This study will: (1) determine the acceptability and feasibility of widespread e-screening in routine prenatal care across different risk subgroups; (2) improve pregnant women’s mental health through increased access to early screening and treatment; (3) inform future trials of e-screening in different perinatal (for example, midwife, obstetrician) and home-based settings; and (4) inform policy development through data regarding the clinical value of psychosocial assessment across varied practice settings and potentially the ease, accuracy, and acceptance of electronic data collection regarding sensitive personal information with pregnant women.

## Trial status

Recruitment will begin in September 2013.

Trial registration: ClinicalTrials.gov Identifier: NCT01899534.

## Abbreviations

ALPHA: Antenatal Psychosocial Health Assessment; CAE: CASI Assessment Evaluation; DES: Disclosure Expectations Scale; EPDS: Edinburgh Postnatal Depression Scale; MINI: MINI International Neuropsychiatric Interview (version 6.0.0); RCT: Randomized controlled trial.

## Competing interests

The authors declare that they have no competing interests.

## Authors’ contributions

DK conceived and designed the study, drafted the grant and the protocol manuscript, will organize and supervise trial implementation, and is responsible for trial management, staff training, and supervision. MPA, AB, KH, GL, GM, SM, SDM, AO, WS, and SVZ participated in writing the grant. MPA, AB, RG, KH, GL, AO, WS, SM, SDM, and SVZ contributed to the study design. KH, GL, SDM, TP, and SVZ participated in study implementation. MLS manages day-to-day trial responsibilities, including supervising staff, monitoring recruitment and data collection, and liaising with recruitment sites. SVZ provides expertise on study methodology and advises on trial management. MPA, KH, GL, and GM provide mental health expertise, and AB and SDM provide obstetrical expertise. AB and SDM provide expertise regarding mental health screening during perinatal care. SM provides statistical and methodological expertise; DK, SM, and RG will conduct statistical analyses; and AO will conduct economic analyses. MLS, DK, KH, and GL will conduct qualitative interviews and DK, GL, KH, MLS, and WS will analyze qualitative data. All authors participated in refinement of the study methods, critically reviewed manuscript drafts, and approved the final manuscript.

## Authors’ information (co-authors listed alphabetically)

DK (PhD) is an Assistant Professor in the Faculty of Nursing and an Adjunct Assistant Professor in the Department of Obstetrics and Gynecology at the University of Alberta, Edmonton, Canada. She holds an Early Career Transition Award through the Alberta Centre for Child, Family, and Community Research. MPA (MD, FRANZCP, MB) is a perinatal psychiatrist and Professor in the Faculty of Medicine at University of New South Wales, Sydney, Australia. She is also the Chair of the Perinatal and Women’s Mental Health Unit at the University of New South Wales, the Director of the St. John of God Mother-Baby Unit in Sydney, Australia, and the lead developer of the *Australian Clinical Guidelines for Perinatal Mental Health* (2011) and the International Marce Society Position Statement on *Psychosocial Assessment and Depression Screening in the Perinatal Period* (2013). AB (MD, CCFP, FCFP) is a family physician in the Mount Sinai Academic Family Health Team and an Associate Professor in the Department of Family and Community Medicine at the University of Toronto, Toronto, Canada. She holds the Ada Slaight and Slaight Family Directorship in Maternity Care in the Ray D. Wolfe Department of Family Medicine at Mount Sinai Hospital in Toronto, Canada. RG (PhD) is a Senior Research Fellow and Clinical Psychologist at the Parenting Research Centre in Melbourne, Australia. KMH (PhD) is a Professor in the Faculty of Nursing and an Adjunct Professor in the Department of Psychiatry at the University of Alberta, Edmonton, Canada. She holds a Canada Research Chair in Stress Disorders in Women. GL (PhD) is an Associate Professor in the Faculty of Nursing at the University of Alberta and is a Certified Psychiatric Nurse. SM (PhD) is an epidemiologist with expertise in statistics, life course analysis, and mental health tool development. She is the senior scientist for the All Our Babies birth cohort study. SDM (MD, FRSCS, MSc) is an Associate Professor in the Division of Maternal-Fetal Medicine in the Departments of Obstetrics and Gynecology, Radiology, and Clinical Epidemiology and Biostatistics. She holds a CIHR New Investigator Award. GM (MD, FRCPC, PhD) is the Vice Dean of the Faculty of Medicine at the University of Calgary, Interim Director of the Mathison Centre for Mental Health Research & Education, and medical lead for the Addiction and Mental Health Strategy (Alberta Health Services), Calgary, Canada; AO (PhD) is a health economist and Associate Professor in the School of Public Health at the University of Alberta, Edmonton, Canada; MLS coordinates and manages the day to day operations of the HOPE (Healthy Outcomes of Pregnancy and Postpartum Experiences) Program of Research, University of Alberta, Edmonton, Canada; WS (PhD) is a Professor in the School of Nursing at McMaster University, Hamilton, Canada. SVZ (MD, PhD) is Director of the Division of Gastroenterology at University of Alberta Hospital (Edmonton, Canada), a Professor in the Faculty of Medicine and Dentistry at the University of Alberta (Edmonton, Canada) and a trial methodologist.
